# Seasonal malaria chemoprevention: successes and missed opportunities

**DOI:** 10.1186/s12936-017-2132-1

**Published:** 2017-11-28

**Authors:** Matthew E. Coldiron, Lorenz Von Seidlein, Rebecca F. Grais

**Affiliations:** 1 Epicentre, 8 rue Saint-Sabin, Paris, France; 2 Mahidol Oxford Tropical Medicine Research Unit, Bangkok, Thailand

**Keywords:** Malaria, Antimalarials, Chemoprevention

## Abstract

Seasonal malaria chemoprevention (SMC) was recommended in 2012 for young children in the Sahel during the peak malaria transmission season. Children are given a single dose of sulfadoxine/pyrimethamine combined with a 3-day course of amodiaquine, once a month for up to 4 months. Roll-out and scale-up of SMC has been impressive, with 12 million children receiving the intervention in 2016. There is evidence of its overall benefit in routine implementation settings, and a meta-analysis of clinical trial data showed a 75% decrease in clinical malaria compared to placebo. SMC is not free of shortcomings. Its target zone includes many hard-to-reach areas, both because of poor infrastructure and because of political instability. Treatment adherence to a 3-day course of preventive treatment has not been fully documented, and could prove challenging. As SMC is scaled up, integration into a broader, community-based paradigm which includes other preventive and curative activities may prove beneficial, both for health systems and for recipients.

## Background

Since its recommendation by the World Health Organization in 2012 [1], Seasonal malaria chemoprevention (SMC) has been introduced in 12 countries [2]. SMC targets children aged 3–59 months in the Sahel during the short, but intense, malaria transmission season. Children are given monthly courses of sulfadoxine-pyrimethamine (SP) and amodiaquine (AQ) for up to 4 months. Each month, AQ is given in daily doses for 3 days, and is given with single-dose of SP on the 1st day. The clinical trials that evaluated strategies similar to what has come to be called SMC used different treatment regimens (SP alone, SP with AQ, SP with artesunate, and artesunate-AQ) with different treatment intervals (monthly versus every 2 months). None gave treatment for more than 3 months of the year [3–9]. With a variety of drug regimens used, direct comparison is difficult, but meta-analysis of clinical trial data showed a 75% decrease in clinical malaria with SMC compared to placebo, a modest impact on the prevalence of anaemia but with high heterogeneity between studies, and no significant decrease in hospitalizations or all-cause mortality, though death rates in all trials were low [10]. The goal of treating all children in the community irrespective of disease is to clear parasites and to provide a prophylactic effect thereafter [11]. Prior to the scale-up of SMC, chemoprevention strategies have been used in health centers in the setting of antenatal visits (IPTp) and routine childhood vaccinations (IPTi) [12, 13].

Since 2012, the scale-up of SMC has been remarkable, with over 12 million children receiving the intervention in 2016. Much of the growth has been driven by a UNITAID-funded consortium, Access-SMC, which provided SMC to 4 million children in 2016 [2]. Other major funders include the World Bank and UNICEF. National Malaria Control Programmes (NMCP) have readily adopted donor-funded SMC, and many non-governmental organizations are supporting its implementation. Few data from programme-implementation settings have been published, but one recent observational study in Mali in 2014 showed that prevalence of parasitaemia at the end of the high transmission seasonal was lower in areas receiving SMC (18%) than in areas not receiving SMC (46%) [14]. For recipients, data from clinical trials suggested high levels of community acceptability when taken during the peak malaria season [15], and at least one study from a programme implementation setting has shown a reasonable cost-effectiveness of the strategy (approximately US $100 per case averted and $3300 per under-5 death averted) [16], but broader data have yet to be published.

Since SMC has been recommended, research on innovative strategies has continued. In an area with long seasonal transmission, children receiving 5 monthly distributions of SMC had approximately 50% lower malaria incidence than children receiving no SMC (or children receiving 3 monthly distributions/ [17]. A trial of azithromycin in addition to SP + AQ has been carried out, and a trial of SMC and seasonal vaccination with the RTS,S/AS01 malaria vaccine is underway [18].

## Challenges for SMC

Despite its impact, SMC remains unsatisfying for several reasons, including its logistical burden, the use of preventive medications that require a 3-day course of therapy, and incomplete coverage of zones which could benefit most from SMC.

SMC distributions occur once per month, necessitating a large commitment of time and resources by NMCPs and their partners. Reaching millions of children every month, every rainy season, is a challenge in any setting, particularly so in the rural Sahel, where infrastructure is poor, and access is difficult especially during the rainy season. As with many preventive interventions, children who stand to benefit the most from SMC (those living farthest from health centres and who have the worst access to care) are also the hardest to reach month after month. With implementing partners facing “SMC fatigue”, it is a challenge to continue reaching these most isolated regions. One way forward could be decentralization, perhaps making one delivery per season, storing enough SP-AQ to cover all four rounds in remote villages, and transferring the responsibility to distribute the anti-malarials to community health workers. Such a strategy would be logistically less challenging but requires close, continued supervision in these areas.

Data from clinical trials have suggested high levels of adherence to the 3-day course required by SMC [19], but to date, there is little objective, quantitative data that confirm adherence (such as plasma drug levels) from areas where SMC is implemented as a routine programme. Poor adherence will accelerate the spread of resistance to SP and AQ, which may in turn lead to decreased protective effectiveness of SMC. Ideally, preventive treatments (and cures) would be single-dose, but no single-dose therapies are available for use in SMC, so ensuring good adherence to the multi-dose treatment is important, both now and for the future. Other regimens have been trialled, notably dihydroartemisinin-piperaquine, which was shown to be non-inferior to SP-AQ in Burkina Faso [20], but still require multiple doses and pose the same problems with adherence.

One of the biggest blank spots in SMC coverage is in northern Nigeria, where an estimated 11 million children live in areas eligible for SMC, but where only approximately 4 million were targeted to receive SMC in 2018. Weak infrastructure and ongoing armed conflicts make programme expansion difficult in this area despite the large potential benefit. One of the few strong systems in that area is related to polio eradication, and one possible entry for SMC into this region could be to repurpose already-existing human resources to also distribute SMC.

## Opportunities

In the Sahel, immunization is administered as a vertical programme in the Expanded Programme on Immunization (EPI). In 2015, at a national level, measles-containing vaccine coverage was 88% in Burkina Faso, 76% in Mali, and 73% in Niger, all below target levels [21]. During the peak season of malnutrition (which frequently overlaps with the malaria season), therapeutic feeding programmes are run as independent vertical programmes by different departments of Ministries of Health. Coverage is chronically low, with only an estimated 7–13% of children suffering from severe acute malnutrition enrolled in a programme [22]. SMC is also managed as another vertical programme by NMCPs. Each of these three large vertical programmes (EPI, nutrition, and SMC) has its own funding streams, with little incentive for integration. A few local pilot programmes, often supported by international partners, have combined SMC with nutritional screening and catch-up vaccination, but these have not been scaled up. Integrating programmes is not only cost efficient for donors, it could also reach more children and increase adherence.

Ideally integrated programmes would be administered through community health workers responsible for many different activities. There is increasing evidence that the existence of a competent community health worker in a village who provides diagnosis and treatment is associated with a reduction of malaria incidence and the prevalence of *Plasmodium falciparum* infections in areas with low malaria endemicity [23]. Although implementing such programmes can be challenging in high endemicity environments [24], taking a more holistic approach to malaria control—and child health in general—in the Sahel could be beneficial. This sort of integrated model may also avoid future problems with sustainability that have been observed in other programmes that involve regular mass distributions of drugs, like those used in several neglected tropical disease programmes, where integrated models have proven both effective and sustainable [25].

## Conclusions

SMC was never intended to be a “magic bullet” [2]. Given the multi-factorial and multi-sectorial nature of malaria control, focus on SMC by potential funders, and even programme participants, highlights the risk of perceiving SMC as a singular solution. As SMC expansion continues, efforts should be made to integrate the programme into a more comprehensive and sustainable community-based paradigm, that also includes other curative and preventive care.

## Abbreviations

AQ: amodiaquine
EPI: Expanded Programme on Immunisation
IPTi: Intermittent preventive treatment for malaria in infants
IPTp: Intermittent preventive treatment for malaria in pregnant women
NMCP: National malaria control programme
SMC: seasonal malaria chemoprevention
SP: sulfadoxine-pyrimethamine
